# Effects of Cinching Force on the Tricuspid Annulus: A Species Comparison

**DOI:** 10.4172/2329-9517.1000283

**Published:** 2017-06-16

**Authors:** Jesus Aleman, Amy Adkins, Lori Boies, Fatima Al-Quiati, Edward Sako, Shamik Bhattacharya

**Affiliations:** 1 School of Science, Engineering, and Technology, St. Mary’s University, 1 Camino Santa Maria, San Antonio, Texas, USA; 2 Department of Cardiothoracic Surgery, University of Texas Health Science Center, San Antonio, Texas, USA

**Keywords:** Cardiac mechanics, Tricuspid valve, Transcatheter repair, Species comparison

## Abstract

**Purpose:**

Tricuspid annuloplasty rings are commonly used to cinch an enlarged tricuspid annulus back to its original shape and size in patients with severe functional tricuspid regurgitation. However, the invasive operation is contraindicated for patients at risk for reoperation. Fortunately, transcatheter repair procedures, currently in the development process, are minimally invasive alternatives to current repair techniques. This study aims to determine the species-dependence of cinching force with the potential of informing transcatheter repair design by quantifying the minimum required cinching force necessary to reduce tricuspid regurgitation.

**Methods:**

The cinching force necessary to reduce the septal-lateral diameter of a dilated annuls was quantified and compared in ten ovine hearts and nine porcine hearts. Additionally, a deparaffinization protocol and Verhoeff-Van Gieson stain were used to compare the microscopic structure of tissue samples at different stages of the experimental procedure in the two species.

**Results:**

The maximum annulus dilation observed for the porcine was 11.2%, and the maximum cinching force was 0.40 ± 0.12 N. As previously demonstrated, ovine hearts yielded a maximum annulus dilation and cinching force of 8.82% and 0.38 ± 0.09 N respectively. Histological stains revealed no gross tissue differences between ovine and porcine septal or free wall tissues.

**Conclusion:**

The cinching force was not species dependent between ovine and porcine models. This study is an essential first step for determining which animal model should be utilized for the development of transcatheter devices.

## Introduction

Functional tricuspid regurgitation (FTR) is a form of heart valve disease that has been linked with morbidity when in conjunction with mitral, aortic, or pulmonary valve complications [1]. FTR commonly occurs as a result of circumferential dilation of the tricuspid annulus. The dilation becomes concerning when coaptation between the three leaflets of the tricuspid valve is insufficient in prevent regurgitation [2–5]. Surgical treatment is often undergone when the annulus dilates to an area greater than 40 mm^2^ [2,6,7]. The treatment options for FTR which include DeVega-suture, Kay bicuspidization, and tricuspid annuloplasty ring require open-heart surgery and are typically done only in adjunct to mitral valve repair [2,6,7]. Currently, the most widespread treatment for FTR is the annuloplasty ring. A tricuspid annuloplasty is used to cinch the enlarged tricuspid annulus back to its original shape and size with the aim of halting regurgitation [8]. Notably, the first annuloplasty operation is low risk for patients who qualify for open heart surgery. However, recurrent regurgitation is seen in 20% of the population and reoperation yields a morbidity rate up to 37% [9,10]. Tricuspid annuloplasty is contraindicated in patients due to the invasive procedure and high risk of reoperation [11]. Fortunately, transcatheter repair procedures such as TRAIPTA, TRIcinch, and Mitraling are in the development process and are minimally invasive alternatives to current tricuspid repair techniques [12–14]. For further development of transcatheter repair techniques, animal studies are necessary to insure efficacy and safety of these treatments.

## Aim

The aim of this study is to determine if cinching force is species-dependent. Specifically, we will 1) compare the cinching force necessary to reduce the area of a dilated annuls and 2) compare microscopic structure of tissue samples at different stages of the experimental procedure in both ovine and porcine models. This study has the potential to inform the minimum cinching force that a transcatheter treatment should produce to reduce regurgitation.

## Materials and Methods

**Figure 1:**
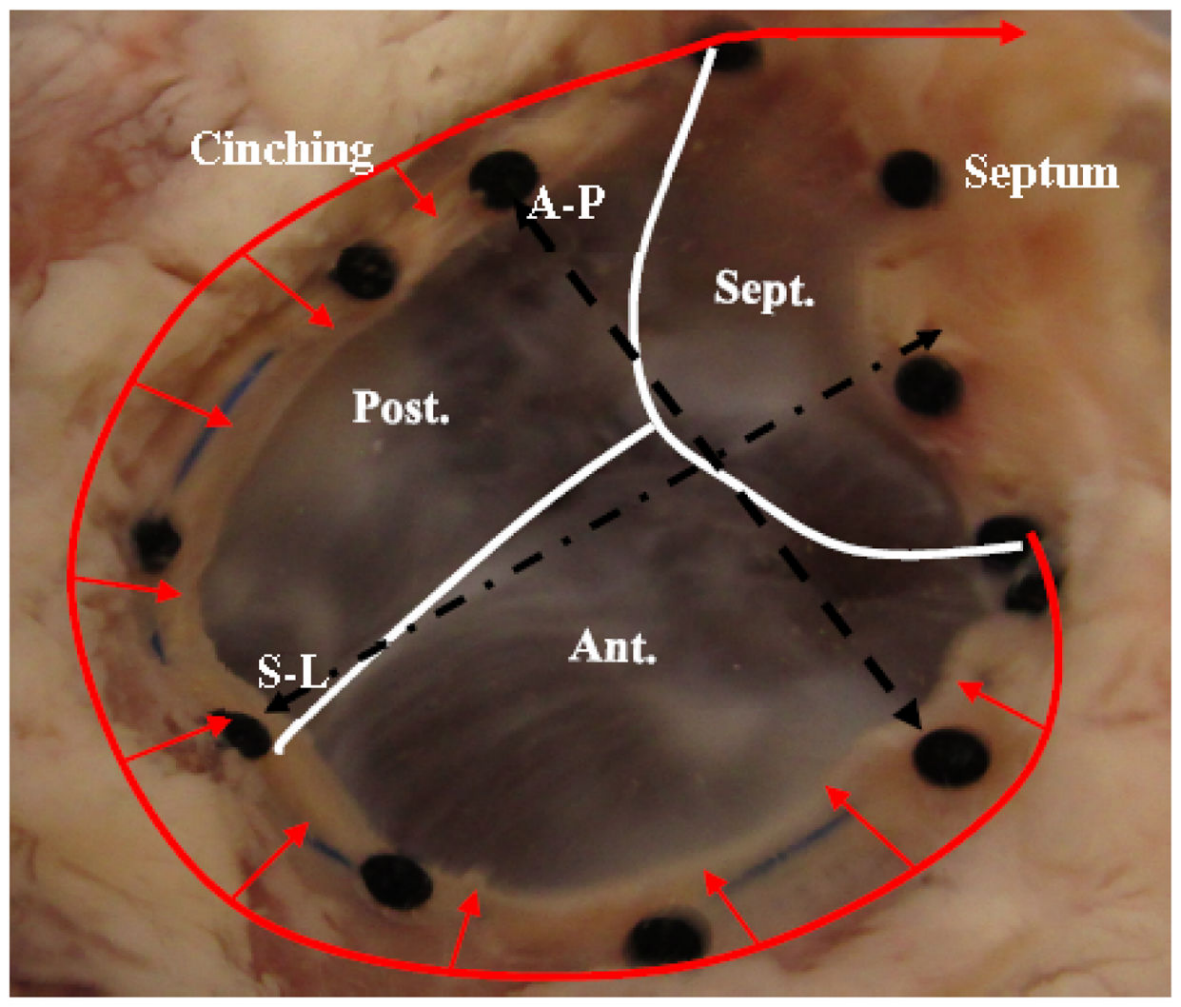
Image of porcine tricuspid valve. Inward arrows show the direction of cinching. Sept.-Septal valve, Post.-Posterior valve, Ant.-Anterior valve, A-P-anterior posterior axis, S-L septal-lateral axis.

Cinching of the annulus-the methodology used to defrost, dilate, cinch, and pressurize the porcine heart was previously established in this lab for the ovine model [15]. The same conditions of non-pressurize (NP) (control), pressurize (P), and dilated-pressurize (DP) were used on the porcine model. When sizing the annulus in the porcine hearts, the T32 sizer was used to normalize the tricuspid annulus (Figure 1). Additionally, the pressure was increased to 32 mmHg due to the size of the porcine hearts. Statistical analysis was conducted to determine statistical differences between models, conditions, and pulling distances using SPSS (version 22.0, IBM).

## Histology

Tissue was isolated, fixed using 10% formalin for 24 hours, washed using physiological bath solution (PBS), and dehydrated using progressive alcohol concentrations. Samples were sent to the UT Health Science Center at San Antonio Histology/Immunohistochemistry Lab to be embedded in paraffin wax, sectioned to a thickness of 5mm and placed onto slides. Once the slides were prepared, they were subjected to a deparaffinization protocol and Verhoeff-Van Gieson stain applied for histological analysis [16].

## Statistical analysis

All measurements are shown as mean ± standard deviation (SD). Two way ANOVAs were used to test for interactions between the two independent variables on each of the five dependent variables. The two factors in this study were pulling distance (0 mm to 24 mm) and condition of the valve (NP, P, DP). The dependent variables were force, area, leakage, septal-lateral diameter, and anterior posterior diameter. In addition, a Tukey post hoc test was used to determine significance for all dependent variables according to pulling distance and condition. A 2-tailed t-test for two samples with unequal variance was used to determine statistical difference in the percent reduction in the septal-lateral diameter divided by force between ovine and porcine hearts. After standardizing the cinching force with the area of the tricuspid valve, a two-sample t-test assuming equal variances was implemented to determine if there was a significant difference between the ovine and porcine models.

## Results

**Figure 2:**
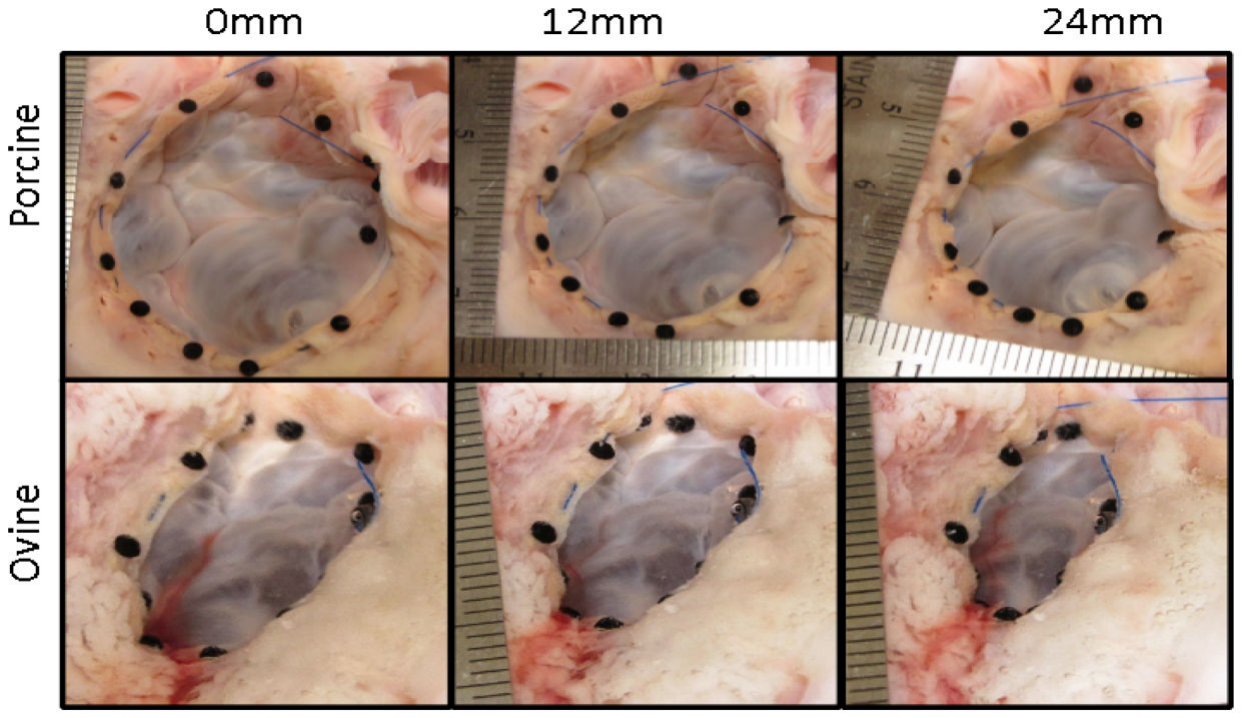
Images of porcine tricuspid valve top and ovine tricuspid valve bottom at pulling distance 0 mm, 12 mm, and 24 mm (left to right).

This study utilized nine porcine hearts and ten ovine hearts. Data is displayed with a mean ± standard deviation. While the pulling distance increased for all three conditions (NP, P, DP), the cinching force increased and the annulus area, anterior posterior, and septal-lateral distances decreased (Figure 2). Reduction in leakage of the porcine specimen was only observed in relation to the pulling distance when the valve was in the DP condition.

**Figure 3:**
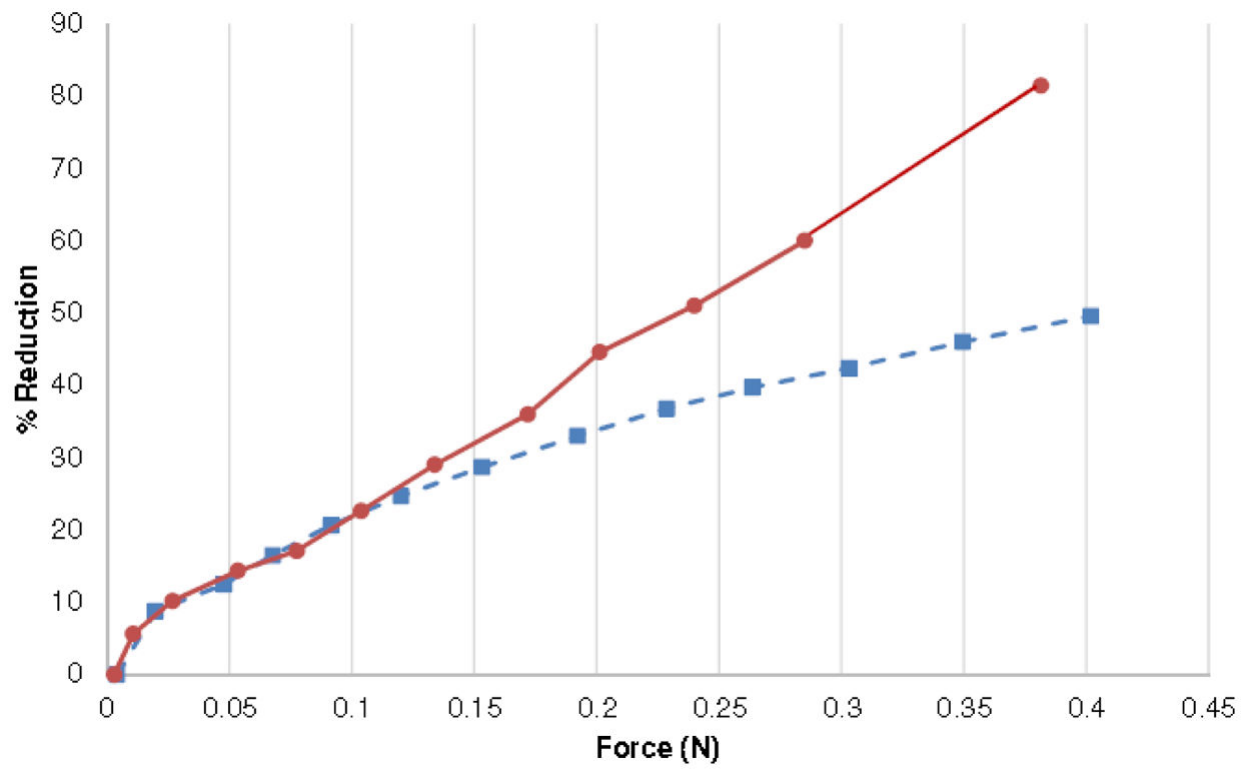
Force vs percent area reduction for the pig heart and the sheep heart.

After chemical dilation by phenol, an increase in annulus area and leakage was observed in both ovine and porcine species. Porcine valve area increased from the P to DP state by 11% (6.5 cm^2^ ± 0.9 cm^2^ to 7.2 cm^2^ ± 0.7 cm^2^) (Figure 3). Leakage increased by 275% (8 L/min ± 5 L/min to 30 L/min ± 10 L/min) with dilation of the valve. Additionally, phenol increased the septal-lateral dimension by 11% (2.8 cm ± 0.3 cm to 3.1 cm ± 0.3 cm) and the anterior posterior dimension by 3.1% (3.2 cm ± 0.3 cm to 3.3 cm ± 0.2 cm). As pulling distance increasing from 0 mm to 24 mm the septal-lateral and anterior posterior diameters decreased in both species. An 18% decrease (2.8 cm ± 0.3 cm to 2.3 cm ± 0.3 cm) was observed in the P condition and 23% (3.1 cm ± 0.3 cm to 2.4 cm ± 0.3 cm) in the DP condition in the porcine specimen. Similarly, the anterior posterior dimension decreased 25% (3.2 cm ± 0.3 cm to 2.4 cm ± 0.3 cm) for the P condition and 27 % (3.3 cm ± 0.2 cm to 2.4 cm ± 0.2 cm) in the DP condition. While the leakage in the DP condition was reduced by 56% (1.8 cm ± 0.7 cm to 0.8 cm ± 0.3 cm) with pulling distance, the leakage in the P condition stayed constant at 0.41 L/min ± 0.05 L/min.

**Figure 4:**
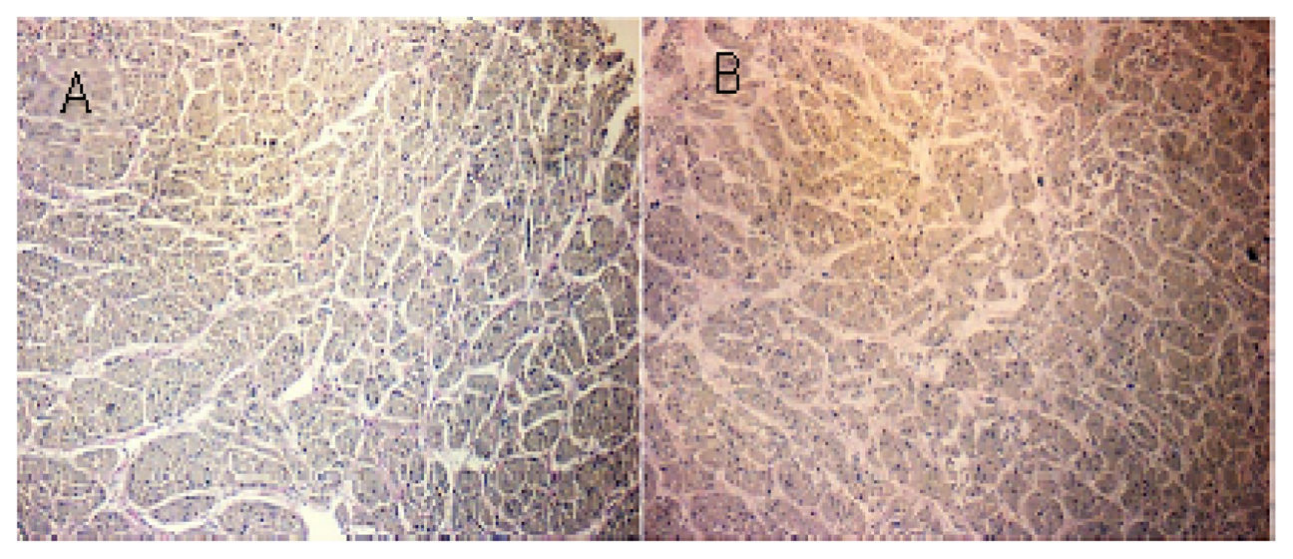
Both sheep (A) and porcine (B) septal tissues were analyzed after both phenol treatment and cinching. Overall there are no gross tissue differences that were observed through the use of elastic stain.

**Figure 5:**
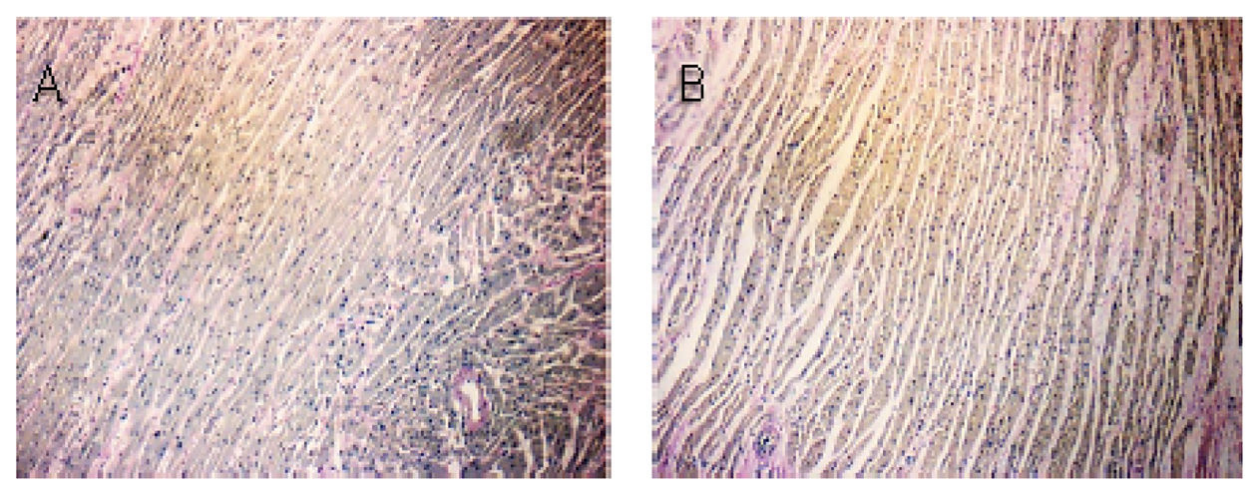
Both sheep (A) and porcine (B) free wall tissues were analyzed after both phenol treatment and cinching. The cardiac muscle tissue found in the free wall of the porcine specimen (B) was found to be in a less compact arrangement than that of the sheep.

**Figure 6:**
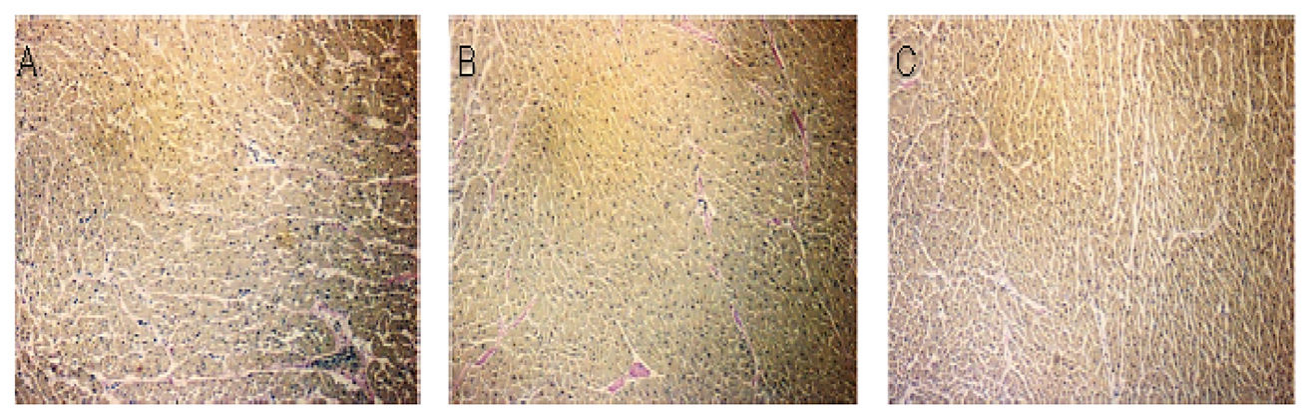
All pictures show sections of the septal wall after cinching treatment. Part A is a sheep septal wall after both phenol treatment and cinching. Parts B and C are from porcine samples where B was not treated with phenol and C was prior to cinching. The tissue shows definite integrity issues when comparing non-phenol treated (B) to phenol-treated (C). When comparing the sheep vs porcine tissue (A and B) there does not appear to be an overt difference in the tissue structure.

Notably, in the ovine specimen a similar pattern was seen with a reduction in leakage of the DP condition with pulling distance but no decrease in leakage in the P condition with pulling distance [15]. Overall, the histological stains revealed no gross tissue differences between ovine and porcine septal or free wall tissues (Figures 4 and 5). The difference in the integrity of tissues that are not cinched, cinched, and are dilated with phenol and cinched can be observed in Figure 6.

## Discussion

In this study, the cinching force required to bring a dilated annulus back to its original size was not dependent on species. The cinching force for the porcine model was determined in the same manner as the ovine model, as a combination of the tissue resistance force and the transvalvular pressure force [15]. The resultant of these two forces is demonstrated in both the P and DP conditions of the valve. The NP condition, in which no transvalvular pressure was applied, represents tissue resistance force. For both the ovine and porcine models, the force due to tissue resistance is significantly smaller (p<0.05) than the transvalvular pressure force at peak systolic pressure. With the addition of phenol to the porcine annulus, an increase in septal-lateral (S-L) diameter of 6.2% was observed as well as an increase of 14.6% to the anterior-posterior diameter (A-P). The overall area showed an increase of 11.2%. This increase in A-P, S-L as well as area is consistent with the dilation seen when phenol was added to the mitral valve of the porcine model [17]. Unlike the ovine hearts where sagging increased the 2D area, the porcine hearts were not observed to have sagging prior to pressurization. This is understood to be a result of the ticker right ventricular wall observed in porcine specimens, supporting the right atrium. The tricuspid annulus’s area increased from NP to P and P to DP condition in the ovine model. From the histological data, we can conclude that there are no overt tissue differences between the porcine and ovine tissue in either the septal wall or free wall of the right ventricle. Notably, it was found that there was no significant difference between the two models (p=0.29) when normalizing the cinching force to the area of the valve. As seen in Figure 2, the percent reduction in area between the pig and sheep for a given cinching force are very similar until approximately 0.1N, where there is diversion. This may be as a result of the larger leakage decrease in the dilated porcine valve as pulling distance was increased. While the ovine leakage was reduced from 82% to 26% of the original dilation, the pig was reduced from 265% to 56% allocating more of the pressure force from the leakage to the valve. For our experiment, ovine and porcine heart models were selected due to having a tricuspid annulus physiologically similar to that of humans. Thus, it can be inferred that the data from porcine and ovine models will have similar cinching forces and reductions in area to humans. To the best of our knowledge, this is the first species comparison study looking at differences in cinching force. Studies have been conducted to determine the biaxial mechanical properties of heart tissues in human, porcine, and ovine models [18]. The study was done on coronary sinus tissue in which a significant difference was found between the animals and humans. In the animal model, tissue was more compliant and provided a lower interaction force than that of the human model. Given that the average human heart was 85 ± 10 years, stiffness observed may be explained by the patients’ elderly age [18], Knowing the difference between animal models is important in the development of new surgical techniques and interventions, which can be effected by the mechanical properties of the tissue. New developments of transcatheter techniques such as TRIcinch, and Mitraling have taken this technique to human tests; however, no data has been produced yet. Both of these methods work by introducing a cinching force inside of the right atrium to reduce the tricuspid area, leading to a decrease in regurgitation and restoration of the coaptation depth of the leaflets [14] TRAIPTA conversely works on the exterior of the heart by placing customized tension along the atrioventricular groove compressing both the tricuspid and mitral valve. While TRIPTA is less invasive than open-heart surgery it still ruptures through the right atrium to extend to the outside of the heart [19].

This study has some limitations that should be addressed. Only two animal models were compared; neither mode was human, thus no direct comparison to human tricuspid valves can be made. This was a quasi-static experiment where we only looked at the 2D area of the annulus in peak systole pressure, meaning that no systemic loading was simulated in the heart. While FTR is commonly diagnosed with an enlargement of the annulus, the 3D area also has an effect on leaflet function. For future studies the cooptation depth would be an important experiment to determine how well treatment bring the leaflets back together as well as conducting this study on human hearts in order to have a direct comparison. However, this study, which was the first study comparing species differences in cinching force, was an essential first step in understanding the minimum required cinching force a transcatheter treatment must produce to reduce regurgitation.
